# Predatory capacity of a shorefly, *Ochthera chalybescens*, on malaria vectors

**DOI:** 10.1186/1475-2875-6-104

**Published:** 2007-08-06

**Authors:** Noboru Minakawa, Kyoko Futami, George Sonye, Peter Akweywa, Satoshi Kaneko

**Affiliations:** 1 Nairobi Research Station, Institute of Tropical Medicine, Nagasaki University, PO Box 19464-00202, NUITM-KEMRI, Nairobi, Kenya; 2 International Center for Insect Physiology and Ecology, Mbita, Kenya

## Abstract

**Background:**

Since *Ochthera chalybescens *had been reported to prey on African malaria vectors, the predatory capacity of adults of this species on *Anopheles gambiae *sensu stricto was explored.

**Method:**

Predatory capacity of this fly on *A. gambiae *s.s. was tested at all developmental stages, including the adult stage in the laboratory setting. Effects of water depth on its predatory capacity were also examined.

**Results:**

This study revealed that *O. chalybescens *preyed on mosquitoes at all life stages except eggs. It was able to prey on an average of 9.8 to 18.8 mosquito larvae in 24 hrs. Mosquito larva size and water depth did not affect predatory capacity. However, the predacious fly preyed on significantly more 2^nd^-instar larvae than on pupae when larvae and pupae were both available.

**Conclusion:**

*Ochthera chalybescens *is, by all indications, an important predator of African malaria vectors.

## Background

Despite the long history of malaria control efforts, malaria remains a major threat to human health. Since malaria vaccine development is slow, and parasite resistance to anti-malarial drugs is developing rapidly, vector control is considered the most practical method for reducing malaria transmission in developing countries [1-3]. Bed nets and indoor residual spray using DDT or pyrethroids have been common measures for malaria vector control [4,5]. However, there is a strong concern about toxicity against other organisms [6] and vector resistance to chemical insecticides [7-10]. Chemical insecticides also kill predators of mosquitoes [11], and the resulting reduction of predators may increase vectors. Predators usually have longer life cycles than their prey [11]. Vector mosquitoes can thus reestablish their population faster after the application of insecticides than their enemies can, and their numbers may become greater than before [12]. Therefore, the use of chemical insecticides requires caution.

Service [11,13] used a serological method to detect predators of *Anopheles gambiae *sensu lato (s.l.) and found that a predacious fly, *Ochthera chalybescens*, is an important predator. Approximately 25% tested positive. Adults of *Ochthera *prey on aquatic invertebrates at the edges of water bodies by using their sickle-shaped front legs [14]. This fly holds its prey between the tibiae and femora of its raptorial forelegs, and it punctures the prey with its labella for consumption. The fly also preys on small winged insects [15]. *Ochthera *is known as a beneficial insect that preys on pest insects such as blackflies and mosquitoes including anophelines [14,16-18]. Larval forms of *Ochthera *are aquatic and also predacious [18-20]. Service [11] claimed that predacious flies, including *O. chalybescens*, play a more important role in reducing populations of *A. gambiae *s.l. in small temporary habitats than in rice fields because the flies are more abundant in small temporary habitats. Since Service [11] had only seen these flies prey on 3^rd^- and 4^th^- instar larvae, pupae, and emerging adults in field, the author argued that the flies cause greater mortality toward the end of the mosquito life cycle.

Although *O. chalybescens *is potentially important as a predator of malaria vectors, no further biological studies have been made since the 1970s [11,13,21]. In this study, the predatory capacity of *O. chalybescens *adults on *A. gambiae *sensu stricto (s.s.) was explored at all developmental stages, including the adult stage, under laboratory conditions. Although Service [11] observed that the predacious fly preys only on older life cycle stages of *A. gambiae *s.l., younger instar larvae may be difficult to see in the field. This study also determined whether water depth affects predatory capacity of *O. chalybescens *because mosquito larvae are able to avoid terrestrial predators by diving [22].

## Methods

### Observation of predatory activity in field and laboratory

Adults of *O. chalybescens *were collected from temporary pools in the Suba district, western Kenya, in July, 2006. *A. gambiae *s.l. larvae inhabited the pools [23,24]. While collecting *O. chalybescens *from the temporary pools, predatory activity was observed to confirm that this fly preys on *A. gambiae *s.l. larvae. In an experimental room at the International Centre of Insect Physiology and Ecology (ICIPE) Mbita Point Research and Training Centre, several of the predacious flies were released into a cage (30 × 30 × 30 cm) and provided a shallow plastic tray (10 × 5 × 2 cm) containing lake water and several *A. gambiae *s.s larvae. The larvae were obtained from the colony maintained at the ICIPE Mbita Point Research and Training Centre. Then predatory activity of *O. chalybescens *was observed for 1 hr.

#### Predation related to prey size and age

The predatory capacity of *O. chalybescens *on eggs of *A. gambiae *s.s. was explored. Twenty eggs were added to each of 40 plastic cups (200 ml size) containing 70 ml water (depth of 2.5 cm). Water was collected from Lake Victoria and filtered prior to the experiment. A wooden stick (2.5 cm long and 0.7 cm wide) was also added to each cup. This stick served as a resting site for the fly. An adult fly was introduced into each of 20 cups of the 40. The other 20 cups without the predacious fly were the control. Flies were kept in cages and starved for 24 hrs prior to the experiment. Each cup was covered with a screen and randomly placed on a table in an experimental room at ICIPE. All cups were covered with a piece of black cloth for 12 hrs after dusk; this reduced the influence of lights in the room. After 24 hrs the remaining eggs in each cup were counted. This procedure was repeated with the other immature stages of *A. gambiae *s.s..

To determine which instar larva (1^st^- to 4^th^-) *O. chalybescens *preys on most, an individual mosquito larva of each instar was introduced into a separate cup to avoid cannibalism [25]. Each 50 ml cup contained 30 ml filtered lake water (depth of 2.5 cm). Seven cups were prepared for each instar (28 cups total: 7 cups × 4 different instars). These cups were randomly placed in a cage, and 2 adults of *O. chalybescens *were released into it. The cage was covered with a piece of black cloth for 12 hrs after dusk. After 24 hrs, each cup was examined for remaining larva. This experiment had 15 replicate cages.

A separate experiment was also designed to examine prey selection of *O. chalybescens*. One 1^st^-instar larva and one 4^th^-instar larva were placed in a cup (200 ml) with 70 ml filtered lake water. Then an adult fly was introduced into the cup and covered it with a screen. Fly activity was observed until either of the two larvae was preyed upon. This procedure was repeated 30 times. This experiment was repeated using one 2^nd^-instar larva and one pupa per cup (20 replicates).

To examine predation on adults, 20 adult females of *A. gambiae *s.s. were released in each of 20 cages (30 × 30 × 30 cm). A starved adult of *O. chalybescens *was then released into each of 10 cages. The other half ten cages, without the predacious fly, served as the control. The cages were randomly placed on a table. All cages were covered with a piece of black cloth for 12 hrs after dusk. After 24 hrs, the live and dead mosquitoes were counted in each cage.

#### Predation related to water depth

Two different water depths were used to evaluate their effects on predatory capacity of *O. chalybescens*. Shallow habitat (depth of 0.5 cm) was created by adding 15 ml of lake water to each of 40 plastic cups (20 ml size). Twenty 3^rd^-instar larvae of *A. gambiae *s.s. were added to each cup, and an adult fly was introduced into each of 20 cups. The remaining 20 cups, without the predacious fly, served as the control. Deep habitat (depth of 2.5 cm) was created by adding 70 ml of lake water to each of 40 cups. Then the same procedure as the above experiments with immature mosquitoes was followed.

### Statistical analysis

The T-test was used to see whether the difference in the number of missing mosquitoes between experimental and control groups was statistically significant. One-way analysis of variance (ANOVA) was used to compare numbers of missing mosquitoes among the four immature life stages. The binomial test was used to examine the difference in predation between 1^st^-instar and 4^th^-instar larvae and between 2^nd^-instar larvae and pupae. Two-way ANOVA was used to test whether the numbers of missing mosquito larvae were statistically different between the two different water depths. The significance level was 5% for all tests.

## Results

### Observation of predatory activity in the field and laboratory

**Figure 1:**
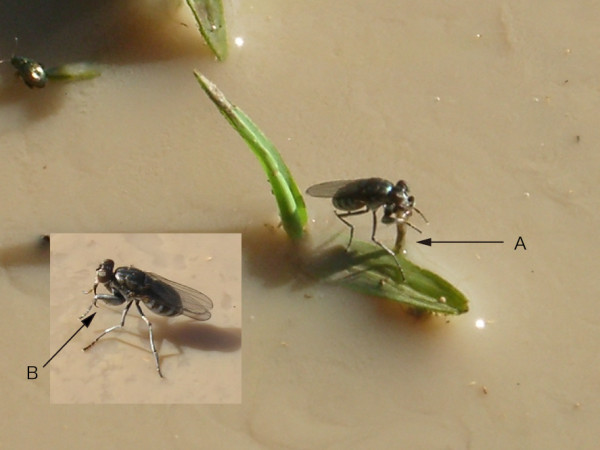
*Ochthera chalybescens *on leaf captured mosquito larva (arrow A) using sickle-shaped front legs (arrow B).

Adults of *O. chalybescens *captured anopheline larvae using their front legs both in the laboratory and in the field. In the field, several adults of *O. chalybescens *on hanging grass leaves at the water's edge (Figure 1). These predacious flies waited until mosquito larvae swam close to them. When a fly aimed at its prey, it tilted its body and extended its forelegs toward the prey. While aiming a fly shook its tarsi. A fly would sometimes fail to catch a mosquito larva and would chase its prey by flying close to the water surface. Adults of *O. chalybescens *also preyed on anopheline and culicine larvae stranded in the mud of drying habitats. In the laboratory setting, adults of *O. chalybescens *preyed on larvae of *A. gambiae *s.s. from the edge of the plastic tray and sticks.

#### Predation related to prey size and age

The number of eggs remained unchanged in all cups with and without *O. chalybescens *(Table 1). However, for all the other immature stages, the average numbers of missing mosquitoes in cups with the predacious fly were significantly greater than in those without a fly (t = 40.21 for 1^st^-instar; t = -21.08 for 2^nd^-instar; t = 13.9 for 3^rd^-instar; t = 8.47 for 4^th^-instar; t = 8.42 for pupae; df = 2, and P < 0.001 for all tests). The average number of missing adult mosquitoes in cages with a predacious fly was also significantly greater than those without (t = 9.97, df = 2, P < 0.001).

**Table 1:** Live, dead and missing mosquitoes of each life stage with and without *Ochthera chalybescens*. Numbers in parentheses are standard deviations.

	Presence				Absence			
		
n = 20	Live	Dead	Live + dead	Missing	Live	Dead	Live + dead	Missing
	
Eggs	20.0 (0.0)	0.0 (0.0)	20.0 (0.0)	0.0 (0.0)	20.0 (0.0)	0.0 (0.0)	20.0 (0.0)	0.0 (0.0)
1st instar	1.2 (2.0)	0.0 (0.0)	1.2 (2.0)	18.8 (2.0)	19.9 (0.5)	0.0 (0.0)	19.9 (0.5)	0.2 (0.5)
2nd instar	2.0 (3.8)	0.2 (0.5)	2.2 (3.8)	17.9 (3.8)	19.8 (0.6)	0.3 (0.6)	20.0 (0.0)	0.0 (0.0)
3rd instar	5.7 (4.1)	0.4 (0.6)	6.1 (4.2)	13.9 (4.2)	20.0 (0.0)	0.0 (0.0)	20.0 (0.0)	0.0 (0.0)
4th instar	5.9 (4.2)	4.4 (2.7)	10.3 (4.3)	9.8 (4.3)	13.4 (3.4)	5.3 (3.5)	18.7 (1.3)	1.3 (1.3)
Pupae	14.4 (2.6)	0.7 (0.7)	15.0 (2.7)	5.0 (2.7)	19.3 (0.7)	0.7 (0.7)	20.0 (0.0)	0.0 (0.0)
Adults	14.2 (2.0)	0.4 (0.5)	14.6 (1.7)	5.4 (1.7)	19.4 (0.8)	0.6 (0.8)	20.0 (0.0)	0.0 (0.0)

When a fly was given four different instar larvae in a cage, mean numbers of missing larvae were 2.7 (SD = 2.6) out of 7 for the 1^st^-instar, 3.0 (SD = 2.0) for the 2^nd^-instar, 2.3 (SD = 2.5) for the 3^rd^-instar, and 2.1 (SD = 2.3) for the 4^th^-instar. The differences in the mean were statistically insignificant (F = 0.52; df = 3, 56; P = 0.673). When *O. chalybescens *was given a choice between 1^st^- and 4^th^-instar larvae, it preyed on 4^th^-instar larva 19 times out of 30. However, the difference in frequency was statistically insignificant (χ^2 ^= 2.16, df = 1, P = 0.142). When a fly was given a choice between 2^nd^-instar larvae and pupae, it preyed on 4^th^-instar larvae 18 times out of 20 occasions, and the difference was significant (χ^2 ^= 14.722, df = 1, P < 0.001).

#### Predation related to water depth

The mean numbers of missing larvae in deep water and shallow water with the fly were 12.2 (SD = 4.9) and 13.9 (SD = 4.2) out of 20 larvae, respectively. Only one larva was missing in one out of 20 cups without the fly in both shallow and deep water. Two-way ANOVA revealed that water depth was not an important variable (F = 1.47, P = 0.230), but that occurrence of the fly was important (F = 324.31; df = 1, 75; P < 0.001). The interaction between water depth and occurrence of the fly was statistically insignificant (F = 1.47; df = 1, 75; P = 1.47).

## Discussion

Missing mosquitoes in cups without predators suggest that cannibalism occurred among mosquito larvae [25]. Averages of 0.3 and 1.3 larvae per cup were missing in the controls for the 1^st^-instar and 4^th^-instar, respectively, and there were no missing larvae in the controls for 2^nd^- and 3^rd^-instars. These results suggest that cannibalism was inconsequential. It is reasonable to conclude that similar numbers of larvae were cannibalized in cups with predators, and thus that nearly all missing mosquitoes (an average of 9.8 to 18.8 per cup) fell prey to *O. chalybescens*.

Although Service [11] did not observe that *O. chalybescens *preyed on young instar larvae of *A. gambiae *s.l. in the field, this study confirmed that the fly preys on all stages of *A. gambiae *s.s. except eggs. The results from the experiments on prey choice among the four larval stages suggest that prey size does not affect predatory capacity. Younger larvae spent more time near the water surface than did older larvae during the experiments, and this behaviour increases the time they are exposed to the predator. Older larvae are able to dive deeper, and diving behaviour is considered a way to avoid predators [22]. However, the larger body size of older larvae may draw more attention from the predator that may offset the shorter time they are exposed near the water surface. Thus, both small and large larvae have some advantages and disadvantages in avoiding predation by *O. chalybescens*.

Although water depth did not affect the fly's predatory capacity in our study, the difference in depth was only 2 cm (0.5 cm vs 2.5 cm). Fourth-instar larvae are able to dive to at least 32 cm [22], so pools deeper than 2.5 cm in the field may be advantageous to larger larvae. The fly would not be able to see mosquito larvae on the bottom of deep habitat.

The results from the experiment with pupae and 4^th^-instar larvae suggest that the predacious fly has more difficulty capturing pupae than larvae. The quickness or the round shape of pupae may account for this. However, the fly may easily prey on mosquitoes during and immediately after emergence as it is also capable of capturing adult mosquitoes. In general, insects are most vulnerable to predation during emergence, and *O. chalybescens *has been observed preying on emerging anopheline mosquitoes in western Kenya [13].

The current results indicate that Service [11,13] underestimated the predatory capacity of *O. chalybescens *on malaria vectors, arguing that the fly preys on only later instar larvae. The same author reported that predacious flies, including *O. chalybescens*, were abundant on small pools and puddles, estimating with photos an average of 226 adult predacious flies per m^2^. A large number of *O. chalybescens *was also seen on the small pools examined in this study. The fly is capable of preying on an average of 9.8 to 18.8 mosquito larvae and 5.0 pupae per day according to this study. Although these figures should not be extrapolated to estimate mortality of mosquitoes by predacious flies in the field, they do indicate that predacious flies are partly responsible for the high mortality (over 90%) of immature *A. gambiae *s.l. and play an important role in controlling a population of malaria vectors [11,13]. This conclusion is more apt for small pools and puddles than for larger water bodies such as pits and rice fields; predacious flies are more abundant in smaller habitats in western Kenya [11].

Small aquatic habitats are short lived due to desiccation [26], which may reduce predators by preventing them from completing their life cycles. It has been suggested that *A. gambiae *s.l. has adapted to small temporary pools by shortening its life cycle to avoid predators [27]. The life cycle of *O. chalybescens *is little known. However, three species of nearctic predacious flies, *O. mantis*, *O. tuberculata*, and *O. exsculpta*, can complete egg to adult development in 15 to 23 days [19]. This implies that *O. chalybescens *completes its development in a similar time span or even faster in its tropical environment. The time span is short enough for the predacious fly to adapt to the productive habitats of *A. gambiae *s.l. [28]. On the other hand, the predacious fly may not necessarily breed in the short-lived habitats where *A. gambiae *s.l. occurs, because the fly is able to fly among habitats.

These characteristics of *O. chalybescens *suggest that it is a better candidate for biological control of African malaria vectors than other organisms. Although a larvivorous fish,*Tilapia zilli*, and a crayfish, *Procambarus clarkii*, have been considered as potential biological control agents for larvae of *A. gambiae *s.l. in Kenya [29-31], their use will not be effective in the small, temporary habitats where *A. gambiae *s.l. usually breeds. They would have to be introduced into each of many small habitats, which would be time consuming and unrealistic. The fish and crayfish might not be able to complete their life cycles in such small habitats; rearing and transporting them would not be cost effective.

## Conclusion

The most important implication of Service's work [11,13] and this study, is the importance of protecting predators of malaria vectors. Direct application of chemical insecticides to aquatic habitats should be avoided because it leads to a rebound in vector populations by eliminating predators [11]. Life cycles of predators are usually longer than that of *A. gambiae *s.l. Application of chemical insecticides to houses and agricultural fields also requires caution; insecticides should not be applied more than necessary. Chemical insecticides may accumulate in aquatic habitats after being carried by rainwater from houses and soil [6], with the side effect of killing beneficial malaria vector predators.

## Authors' contributions

All authors participated in the study design, and carried out the field and laboratory experiments. NM conceived the study, performed the statistical analysis, and drafted the manuscript. All authors read and approved the final manuscript.
